# New Introductions, Spread of Existing Matrilines, and High Rates of Pyrethroid Resistance Result in Chronic Infestations of Bed Bugs (*Cimex lectularius* L.) in Lower-Income Housing

**DOI:** 10.1371/journal.pone.0117805

**Published:** 2016-02-22

**Authors:** Ronald W. Raab, Julia E. Moore, Edward L. Vargo, Lucy Rose, Julie Raab, Madeline Culbreth, Gracie Burzumato, Aurvan Koyee, Brittany McCarthy, Jennifer Raffaele, Coby Schal, Rajeev Vaidyanathan

**Affiliations:** 1 College of Integrated Science and Technology, James Madison University, Harrisonburg, Virginia, United States of America; 2 SRI International, Center for Immunity and Infectious Diseases, Harrisonburg, Virginia, United States of America; 3 Department of Entomology, North Carolina State University, Raleigh, North Carolina, United States of America; 4 Harrisonburg High School, Harrisonburg, Virginia United States of America; University of Cincinnati, UNITED STATES

## Abstract

Infestations of the common bed bug (*Cimex lectularius* L.) have increased substantially in the United States in the past 10–15 years. The housing authority in Harrisonburg, Virginia, conducts heat-treatments after bed bugs are detected in a lower-income housing complex, by treating each infested unit at 60°C for 4–6 hours. However, a high frequency of recurrent infestations called into question the efficacy of this strategy. Genetic analysis using Bayesian clustering of polymorphic microsatellite loci from 123 bed bugs collected from 23 units from May 2012 to April 2013 in one building indicated that (a) 16/21 (73%) infestations were genetically similar, suggesting ineffective heat-treatments or reintroductions from within the building or from a common external source, followed by local spread of existing populations; and (b) up to 5 of the infestations represented new genotypes, indicating that 5 new populations were introduced into this building in one year, assuming they were not missed in earlier screens. There was little to no gene flow among the 8 genetic clusters identified in the building. Bed bugs in the U.S. often possess one or both point mutations in the voltage-gated sodium channel, termed *knockdown resistance* (*kdr*), from valine to leucine (V419L) and leucine to isoleucine (L925I) that confer target-site resistance against pyrethroid insecticides. We found that 48/121 (40%) bed bugs were homozygous for both *kdr* mutations (L419/I925), and a further 59% possessed at least one of the *kdr* mutations. We conclude that ineffective heat treatments, new introductions, reintroductions and local spread, and an exceptionally high frequency of pyrethroid resistance are responsible for chronic infestations in lower-income housing. Because heat treatments fail to protect from reintroductions, and pesticide use has not decreased the frequency of infestations, preventing new introductions and early detection are the most effective strategies to avoid bed bug infestations in multistory apartment buildings.

## Introduction

The common bed bug, *Cimex lectularius* L., is an obligate, blood-feeding ectoparasitic insect that avidly feeds on humans. Global bed bug infestations were common through the 1940s, when inorganic pesticides were used to control bed bugs and other urban pests. From World War II until the 1960s, DDT (dichlorodiphenyltrichloroethane) proved to be an inexpensive, toxic, and effective residual treatment against many arthropods including bed bugs, and bed bugs were largely eliminated from human habitations in the United States [1–4]. In the past two decades, however, populations of *C*. *lectularius* have increased in North America, Europe, and Australia [5, 6]. Industry surveys report a hundred-fold increase in calls for bed bug control, and almost all pest control operations currently respond to bed bug problems [7]. Explanations for this resurgence include increased international travel, changes in pest control practices for other urban pests, increased trade in secondhand furniture, and widespread bed bug resistance to commercially-available pesticides registered for use against this insect [8–10].

Although many bed bug population management and eradication tactics have emerged in the last decade, the two most common and effective approaches are residual insecticides and thermal intervention. The efficacy of residual insecticides has been severely compromised, however, by the rapid evolution of resistance against these commonly used classes of chemistry. Bed bug resistance to DDT was first identified in the 1940s at the Naval Base in Pearl Harbor and to DDT and lindane (γ-hexachlorocyclohexane) globally in the 1950s [11–13]. Resistance to the organophosphate insecticides malathion and fenthion was detected by 1970 [14, 15]. By 2007, resistance against two pyrethroid insecticides, deltamethrin and λ-cyhalothrin, was detected in bed bugs from Kentucky and Ohio [8]. Two point mutations in the α-subunit of the voltage-gated sodium channel of resistant bed bugs result in a valine to leucine substitution (V419L) and a leucine to isoleucine substitution (L925I) [16]. A survey revealed that 88% of bed bug populations collected from 17 states across the U.S. possessed one or both of these *knockdown resistance* (*kdr*) mutations, which conferred target-site resistance against deltamethrin and related pyrethroid insecticides [17]. In addition to *kdr*, subsequent studies revealed multiple mechanisms of pyrethroid resistance in bed bugs, including increased expression of cytochrome P450 monooxygenases, glutathione *s*-transferases, and carboxylesterases, enzymes essential for the detoxification of insecticides [18–20].

Arguably the most effective strategy to eliminate a bed bug infestation is thermal intervention, whereby the infested space is heated to 60°C and maintained at this temperature for at least 4 hours [21, 22]. A major advantage of this approach is its thoroughness in eradicating all bed bugs (and other insect pests) in a single remediation without the use of pesticides. However, major limitations of heat-treatments include their much higher cost compared to other strategies, the lack of residual efficacy to prevent re-infestation, and the requirement for specialized equipment and training. Moreover, because thermal interventions in individual units of apartment complexes are often conducted sequentially or on an “as needed” basis, bed bugs from untreated units can readily reinfest heat-treated units.

Population genetics is central to understand the spread of insecticide resistance traits and bed bug reinfestation after intervention. Two population genetic studies of bed bugs suggest that many infestations are started by small propagules, as small as a single mated female and/or her progeny [23, 24]. Booth et al. [23] developed high-resolution microsatellite markers to investigate the dynamics of introduction and spread within a single apartment building. Bed bug collections within each apartment were characterized by high levels of relatedness and low levels of genetic diversity, suggesting that infestations originated with a single mated female or her progeny, or by a female mated with closely related males [23, 24]. The bed bug genotypes suggested that infestations in one apartment building started from a single introduction followed by extensive spread, whereas in two other buildings there were two or more unique introductions followed by spread [23]. Thus, extinction of some introduced genotypes notwithstanding, unique introductions into buildings appear to be rare. The high rate of inbreeding within a matriline may also select for the persistence of *kdr* mutations. In contrast, bed bug collections from across the Eastern U.S. indicated high genetic diversity and differentiation among infestations, suggesting multiple unique introductions of bed bugs [24].

A five-story apartment complex in Harrisonburg, Virginia, offered a unique opportunity to investigate the spatial and temporal changes in bed bug populations in response to thermal interventions. By genotyping bed bugs from multiple units over multiple collection events, we sought to determine whether bed bugs were escaping and hiding during the thermal treatment and returning to reinfest the same apartment and its neighbors, or if subsequent re-infestations represented new, unique introductions. We compared polymorphic microsatellite loci from bed bugs collected over a year from this building to determine if chronic reinfestations were the product of the same matriline that escaped the treatment and spread from unit to unit or if each infestation was a new introduction from elsewhere. In addition, because we observed extensive pesticide use by tenants, and pyrethroid resistance is widespread in the U.S., we assessed the frequency of *kdr* mutations to infer the extent of pyrethroid resistance.

## Materials and Methods

### Bed Bug Collection and Sample Preparation

Our study site was a five-story, Section 8 New Construction Project originally built in 1983 with poured concrete and blocks commonly known as concrete masonry units (CMU); walling contained no wood including drywall, and universal chases ran vertically between rooms from the ground to the top floor to accommodate plumbing and electrical wiring (M. Wong, personal communication). The annex to the building was built in 1993 of poured concrete, CMU, and pressed wood drywall. The entire building housed 120 one-bedroom units for the elderly, persons with disabilities, and the working poor. Most of the residents earned at most 60% of the area median income, with many individuals earning 30% or less [25]. Bed bugs were collected 41 times from May 2012 to April 2013 from this building. Couches, recliners, bedsheets, mattresses, and box springs in each unit were inspected for 15 to 45 minutes, depending on the presence of bed bugs. Collections from May to September 2012 were pooled for each apartment, but individual bed bugs from each unit from October 2012 to April 2013 were processed for *kdr* and microsatellite analysis (*n* = 123). Individual bed bugs collected from May to September 2012 were included in the *kdr* analysis. Each collection of bed bugs was followed by a heat-treatment of the infested unit by professional pest control technicians, which entailed subjecting each unit to 60°C convective heat for 4–6 hours [25]. Follow-up inspections were conducted for those units that reported subsequent infestations.

We also collected 9 bed bugs from a hotel 24 km from the apartment building and 3 bed bugs from a homeless shelter 1.8 km from the apartment building. This study was possible because of the cooperation of residents and managers who allowed us to collect samples in their homes and hotel rooms.

Bed bugs were preserved immediately in 90% ethanol and stored at -20°C until genomic DNA (gDNA) was extracted using a DNeasy blood and tissue kit (Qiagen, Valencia, CA) following the manufacturer’s supplementary protocol for insect DNA extraction. Concentrations of gDNA were calculated (Nanodrop, Thermo Scientific, Waltham, MA), and quality was assessed on 1% agarose gels stained with ethidium bromide.

### Amplification of Knockdown Resistance Genes

To amplify the α-subunit of the voltage-gated sodium channel that contains the 419 and 925 *kdr* mutations, 1 μL of bed bug gDNA was used as a template with 5 μL of Fast Cycling PCR 2x master mix (Qiagen), 1 μL of Fast Cycling PCR dye (Qiagen), 0.5 μL each of forward and reverse primer (100 ng/μL), and 2 μL of sterile water. Primer sequences are listed in Table 1. PCR conditions included an initial denaturation of 5 min at 95°C and 29 rounds of [94°C for 5 sec, 62°C for 5 sec, and 68°C for 5 sec], with a final holding temperature of 7°C. The expected size for either of the “419” fragments was 256 basepairs, and the expected size for either of the “925” fragments was 411 basepairs. Polymerase chain reaction (PCR) results for each bed bug were resolved by standard 1% agarose gels to determine presence or absence of the wildtype or *kdr* mutation and to determine homozygosity or heterozygosity of each allele.

**Table 1:** Primers used to amplify *kdr* genes.

V419 Forward	5’-ATATCCCTGGGATCATTCTACCTCG-3’
L419 Forward	5’-ATATCCCTGGGATCATTCTACCTCC-3’
419 Reverse	5’-TGATGGAGATTTTGCCACTGATG-3’
L925 Forward	5’-ATTATGGGCAGAACAGTGGGTGCCCC-3’
I925 Forward	5’-ATTATGGGCAGAACAGTGGGTGCCCA-3’
925 Reverse	5’-TGCCTATTCTGTCGAAAGCCTCAG-3’

### Microsatellite Analysis

Genetic analysis was performed on eight microsatellite loci (*BB38B*, *BB31B*, *Clec11*, *Clec6*, *BB15b*, *Clec48*, *Clec21*, *BB28b*) developed by Booth et al. [23]. We followed PCR conditions of these authors [23] with the forward primer in each primer pair end-labeled with M13F-29/IRD700 or 800 IRDye tag (Li-Cor Biosciences, Lincoln, NE). After addition of formamide, amplified products were denatured at 95°C for 4 min before loading on a 6% polyacrylamide gel and run on a Li-Cor 4300 automated DNA sequencer. Fluorescent-labeled size standards (50–350 IRDye) were loaded on the gel for accurate product sizing. Genotypes were scored using the GeneProfiler software (Scanalytics, Inc., BD Biosciences Bioimaging, Rockville, MD).

We conducted tests of genotypic differentiation for all pairs of populations (sample collections) using the exact χ^2^ test implemented in the online program GenePop [26, 27]. Only collections with 3 or more individuals were used in these calculations. *F*-statistics and degree-of-relatedness were estimated using the program FSTAT version 2.9.3.2 [28]. Partial Bayesian cluster analysis of predefined groups (sample collections) was performed using BAPS 6.0 [29, 30] with the aim of identifying the optimum number of genetic clusters among groups of collections with an upper bound of 14 populations. BAPS clusters individuals into genetically distinct groups that differ in allele frequencies. All individuals were included in this analysis, even samples consisting of a single individual. To determine the proportion of individuals within a cluster showing significant evidence of admixture, we followed the approach of Herborg et al. [31] and used the ‘nonequilibrium’ method of individuals-based admixture as implemented in BAPS. For the minimum size of a population, we used 3 individuals with 50 iterations, and we used the default-value of 50 for the number of reference individuals per population and 10 iterations per reference individual. We set the critical value for admixture at a posterior probability of 0.05. We then used the Plot Gene Flow function in BAPS to create a network diagram showing the relative amount of ancestry among individuals in each cluster.

## Results and Discussion

Chronic bed bug infestations in this multistory apartment building appear to be the consequence of (a) single introductions expanding and spreading to neighboring units; (b) bed bugs escaping heat treatments and rapidly reinfesting the same units that had been heat-treated; (c) new introductions from outside; and (d) a high frequency of *kdr* mutations that confer resistance to pyrethroids, the insecticides most commonly used to control these pests.

In the first six months of 2012, housing authorities in Virginia spent $404,364 to control 1,047 infestations, representing 6.3% of all housing units in the Commonwealth [25]. Most of the infestations in Harrisonburg were in units dedicated to the elderly and persons with disabilities, who were more likely to lack the resources to detect and control chronic infestations. The Harrisonburg Redevelopment and Housing Authority spends $800 to heat-treat one unit, but bed bugs appear to disperse to adjacent units and reinfest the treated unit, indicating that this is not an effective, long-term strategy as currently implemented in this apartment building.

We conducted a building-wide collection of bed bugs in June 2012 and subsequent broad collections in September/November 2012 and February/April 2013. We thus verified that apartments adjacent to infested units were uninfested before heat-treatments. Because a room gradually warms to 60°C over the course of 3 hours before the actual 4–6 h treatment, some bed bugs in the walls and furniture likely escape or hide in harborages to avoid the heat. We think that bed bug infestations in poured concrete apartment buildings might be especially difficult to control because walls, floors and ceilings are poor conductors of heat, and cold exterior walls may exacerbate this problem in winter. We also observed that tenants who evacuated their units to accommodate heat-treatment visited friends in other units, possibly transferring bed bugs or their eggs in the process. We think this explains how genetically similar bed bugs suddenly appear in units on different floors or in another part of the building.

### Frequency of *kdr* in Field Populations

A recent study found that 88% of bed bug populations in the U.S. possess target-site resistance against pyrethroids, and the *kdr* mutations were associated with significant resistance to deltamethrin, a pyrethroid insecticide commonly used to control bed bugs [17]. Populations were classified into four qualitative genotypes named “A-D”. Genotype “A” possesses V419 and L925 and is susceptible to pyrethroids. “B” possesses I925 only and confers some resistance, but the role of 419 is unknown. “C” possesses L419 and I925 and is highly resistant to pyrethroids. “D” possesses L419 only and has not been assessed for phenotypic resistance [17].

**Fig 1:**
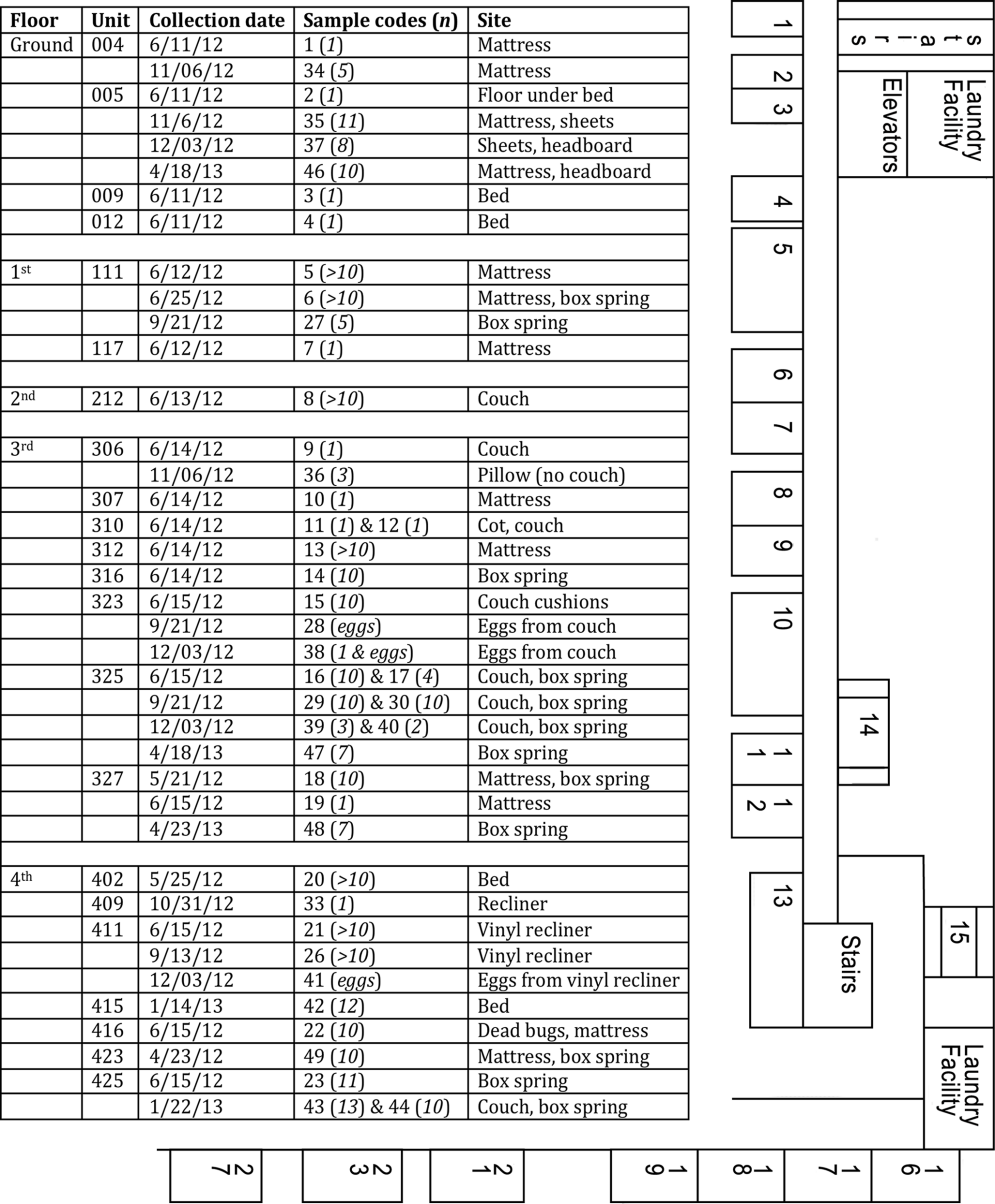
Units and dates in an apartment complex from which bed bugs were collected from May 2012 to April 2013. Consecutive sample numbers 11 & 12, 16 & 17, 29 & 30, 39 & 40, and 43 & 44 represent two sites in the same apartment from which bed bugs were collected on the same date. The number of bed bugs in each sample (*n*) is provided after the sample number. Sample 24 was an internal laboratory colony and is not shown. Samples 31 and 32 were collected at a hotel and homeless shelter, respectively, and were used as outgroups. Because all five floors have the same layout, a common floorplan is provided to help visualize the proximity of units to one another.

**Fig 2:**
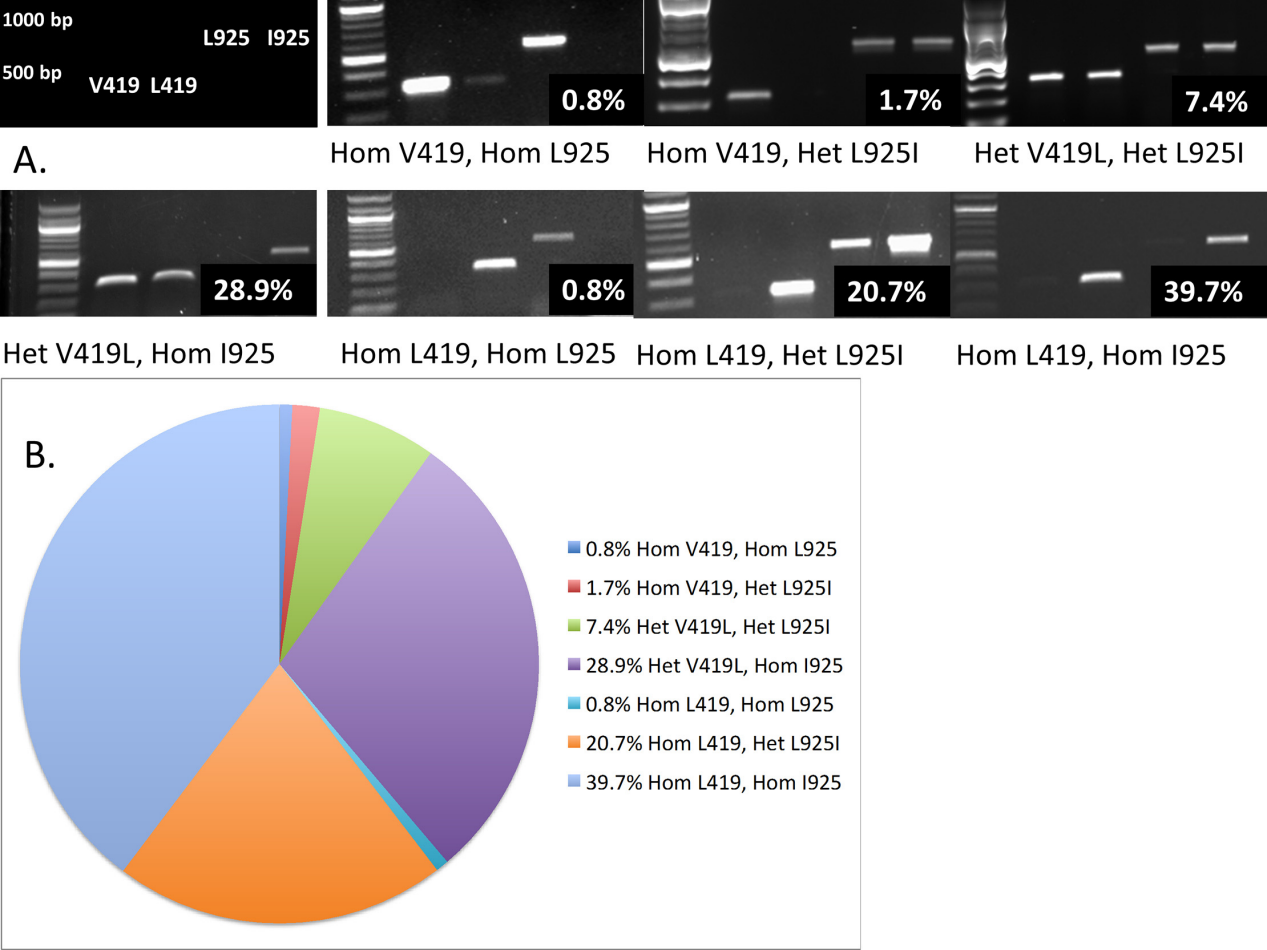
*kdr* Diploid genotypes following PCR amplification of apartment samples. (A) The frequency of each genotype is presented in the bottom right corner of each gel image. The two brightest bands in the molecular weight ladder denote 500 and 1000 basepairs. “Hom” denotes homozygous, and “Het” denotes heterozygous. Two genotypes–Hom V419/Hom I925 and Het V419L/Hom L925 –were not represented in any of the collections and are therefore not shown. (B) Frequency of 419 or 925 susceptible (V419/L925) or *kdr* mutant (L419/I925) alleles from bed bugs collected in an apartment complex.

Fig 1 presents the units and dates in the apartment complex from which bed bugs were collected from May 2012 to April 2013. Examples of all identified *kdr* genotypes are presented in Fig 2A. We found that 120/121 bed bugs collected in this apartment complex possessed one or both mutations associated with pyrethroid resistance. Forty-eight of 121 (40%) bed bugs collected were double homozygous for the *kdr* mutations L419 and I925 (Table 2, Fig 2B). Nine bed bugs were heterozygous for both V419L and L925I. Almost all of the remaining bed bugs were heterozygous for one mutation (V419L or L925I) and homozygous for the other (L419 or I925). Only 1 bed bug (0.8%) collected in apartment 005 in November 2012 was homozygous for both wild-type alleles (V419/I925).

**Table 2:** Frequency of *kdr* genotypes from bed bugs collected from individual apartment units.

Unit	Collection date (d/mo/yr)	Heat-treatment date	Sample code	Item	HomV419/HomL925	HomV419/ Het L925I	HomV419/ HomI925	HetV419L/ HomL925	HetV419L/ HetL925I	HetV419L/ HomI925	HomL419/ HomL925	HomL419/ HetL925I	HomL419/ HomI925
004	6/11/12	11/6/12	1	mattress									1
004	11/6/12	11/6/12	34	mattress						1		2	2
005	6/11/12	6/25/12	2	bed								1	
005	11/6/12	11/6/12	35	blanket/ bed	1	1				1		6	4
005	12/3/12	12/10/12	37	blanket/ bed								2	6
005	4/18/13	4/22/13	46	mattress					3	1		3	2
117	6/12/12	8/8/12	7	bed					1				
306	6/14/12	6/25/12, 8/9/12	9	couch					1				
306	11/6/12	11/6/12	36	pillow									
307	6/14/12	6/25/12	10	mattress								1	
310	6/14/12	3/16/12, 5/1/12	11	cot									1
310	6/14/12	3/16/12, 5/1/12	12	couch								1	
323	12/3/12	12/7/12	38	couch						1			
325	12/3/12	12/7/12	39	bed							1	2	
325	12/3/12	12/7/12	40	couch		1							1
325	4/18/13	4/22/12	47	box spring						4			4
327	6/15/12	6/25/12	19	mattress					1				
327	4/23/13	3/4/2013	48	box spring								1	6
409	10/31/12	4/24/12, 8/8/12	33	recliner									1
411	12/3/12	6/25/12, 9/19/12	41	vinyl recliner									1
415	1/14/13	1/14/13	42	mattress					1	2		2	7
423	4/23/13	4/26/13	49	bed						2		3	5
425	1/22/13	1/17/13	43	couch						7			6
425	1/22/13	1/17/13	44	box spring					1	6		1	1
427	2/4/13	2/14/13	45	box spring					1	10			
				**SUM**	1	2	0	0	9	35	1	25	48
				**Percent**	0.8	1.7	0.0	0.0	7.4	28.9	0.8	20.7	39.7

We detected multiple *kdr* genotypes on different dates from the same apartment (Table 2). Using apartment 005 as an example, in June 2012 we collected one bed bug that was L419/L925I; out of thirteen bed bugs collected in November 2012, we identified one V419/L925, one V419/L925I, one V419L/I925, six L419/L925I, and four L419/I925; in December 2012 we identified two L419/L925I and six L419/I925; and in April 2013 we identified three V419L/L925I, one V419L/I925, three L419/L925I, and two L419/I925.

We also detected multiple *kdr* genotypes from collections made on the same date but from different sites in one apartment. For example, we collected 13 bed bugs from a couch and nine from a box spring in apartment 425 in January 2013. The samples from the couch yielded seven V419L/I925 and six L419/I925, whereas the samples from the box spring yielded one V419L/L925I, six V419L/I925, one L419/L925I, and one L419/I925 (Table 2). In addition, we noticed heterogeneity in genotype distribution from bed bugs collected from a cot and couch in unit 310 in June 2012 and a bed and couch in unit 325 in December 2012.

Although a correlation has been described between the four major *kdr* genotypes (A-D) and resistance ratios [17], the resistance phenotypes of the other seven genotypes have not been described. Moreover, no experimental studies have correlated specific genotypes to a quantifiable level of pyrethroid resistance. Genetic crosses to obtain specific genotypes would be needed to assay against pyrethroids and relate *kdr* genotypes to insecticide resistance ratios.

### Microsatellite Results and Cluster Analysis

Many of the samples were genetically differentiated from other samples according to the χ^2^ exact test (Table 3). Samples collected at the same time from different locations in the same apartment unit were not significantly differentiated and were therefore pooled in calculating allele numbers and heterozygosity. Two pairs of samples—39 & 40 and 43 & 44—fell into this category. The number of alleles per locus across the samples collected from the apartment building with at least three individuals present ranged from 1–9 with a mean of 4.6 (Table 4). Considering all samples, including those from a homeless shelter (sample 32) and a hotel (sample 31), the number of alleles per locus ranged from 2–9. The number of alleles per locus within sample locations was much lower, averaging less than three in all cases, and less than 2 for all samples except samples 35, 42 and 49. With the exception of samples 42 and 49, which contained a maximum of six and five alleles at a locus, respectively, all samples contained at most 3 alleles at any one locus, and six of the 13 samples had at most two alleles per locus (samples 31, 39 & 40, 43 & 44, 45, 46, and 48).

**Table 3:** Genetic differentiation among samples at microsatellite loci as determined by χ^2^ exact tests.

		Samples ^a^												
Samples		31	34	35	36	37	39	42	43	44	45	46	47	48
	34	++												
	35	++	-											
	36	++	++	++										
	37	++	+	0	++									
	39	++	0	0	+	-								
	42	++	+	++	++	++	-							
	43	++	+	++	++	++	-	++						
	44	++	+	++	++	++	0	++	0					
	45	++	+	++	++	++	0	+	-	0				
	46	++	+	++	++	++	-	++	+	+	0			
	47	++	-	+	++	+	0	+	+	-	0	-		
	48	++	-	+	++	+	0	+	+	-	0	-	0	
	49	++	+	+	++	++	0	+	+	-	-	+	0	-

^a^ Sample 31 came from a hotel. Samples 34–49 came from the same apartment building.

“++” denotes two highly differentiated sample collections at a *p*-value < 10^−7^; “+” denotes two sample collections differentiated at a *p*-value < 0.01; “-”denotes two sample collections that are not significantly differentiated at a *p*-value > 0.01; and “0” denotes two identical populations at a *p*-value > 0.1.

**Table 4:** Number of microsatellite alleles present in each sample.

	Sample number ^a^														
Locus	31^b^	34	35	36	37	39 & 40	42	43 & 44	45	46	47	48	49	Total	Total Apartments only
*BB38B*	2	2	3	1	2	1	2	2	1	1	3	1	1	6	5
*BB31B*	2	3	3	3	2	2	1	1	1	1	1	2	2	7	7
*Clec11*	2	2	3	2	2	2	2	2	2	2	2	2	3	3	3
*Clec6*	1	2	1	1	1	1	1	1	1	1	1	1	2	2	2
*BB15b*	2	2	3	1	2	1	3	1	2	2	2	2	5	9	8
*Clec48*	1	1	2	1	1	1	1	1	1	1	1	1	2	2	2
*Clec21*	1	1	1	1	1	1	1	1	1	1	1	1	1	2	1
*BB28b*	1	2	3	1	3	1	6	2	2	1	1	1	3	9	9
Mean	1.5	1.875	2.375	1.375	1.75	1.25	2.125	1.375	1.375	1.25	1.5	1.375	2.375	5	4.625

^a^ Samples from different locations within the same apartment collected on the same date were pooled.

^b^ Sample 31 came from a hotel. Samples 34–49 came from the same apartment building.

**Fig 3:**
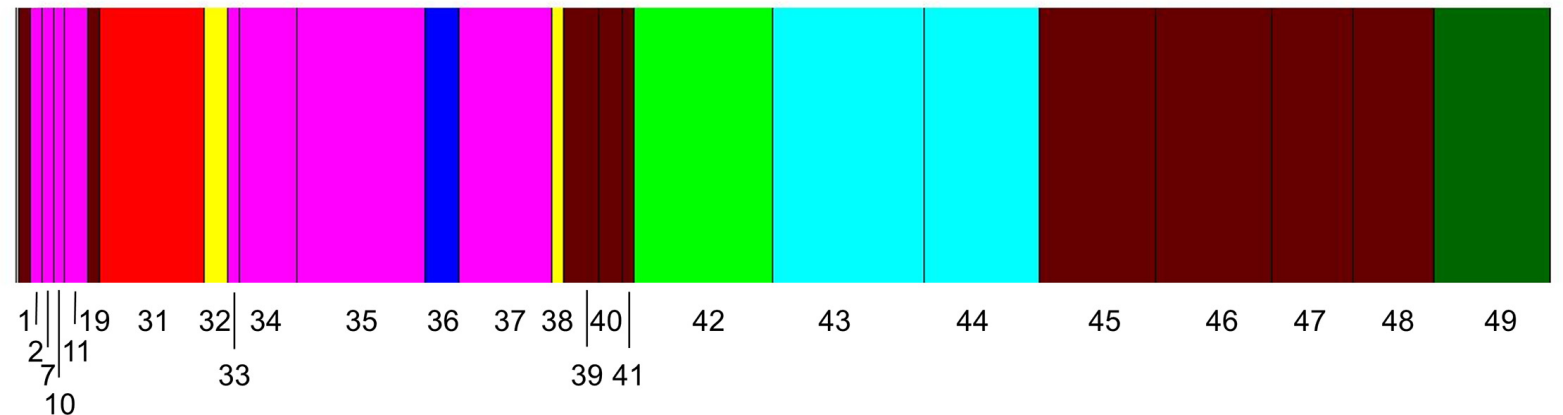
Genetic clustering of samples using the Bayesian method implemented in the program BAPS. The numbers refer to the sample numbers. The width of each band is proportional to the number of individuals in each sample. Samples collected at a hotel (sample 31) and homeless shelter (sample 32) are included in the analysis.

Bayesian clustering analysis of all samples, including those collected at a nearby hotel (sample 31) and homeless shelter (sample 32), identified eight clusters (Fig 3), corresponding to eight genetically distinct populations. The samples collected at the hotel and homeless shelter formed two distinct clusters, but interestingly, sample 38 (apartment 323 collected in December 2012) also clustered with the homeless shelter sample. However, sample 38 was comprised of a single individual and eggs, raising some doubts about the robustness of this assignment. When analyzed by cluster, the number of alleles per cluster was similar to that per sample site, with a maximum of 3 alleles per locus (Cluster 3) and four clusters with two or fewer alleles per locus on average (Table 5).

**Table 5:** Number of microsatellite alleles present in each of the genetic clusters identified in BAPS.

	No. of alleles by cluster								
Locus	Cluster 1	Cluster 2	Cluster 3	Cluster 4	Cluster 5	Cluster 6	Cluster 7	Cluster 8	Total
*BB38B*	2	3	3	1	3	2	2	1	7
*BB31B*	2	3	4	3	4	1	1	2	9
*Clec11*	2	2	3	2	2	2	2	3	3
*Clec6*	1	1	2	1	1	1	1	2	2
*BB15b*	2	3	4	1	2	3	1	5	10
*Clec48*	1	2	2	1	1	1	1	2	2
*Clec21*	1	2	1	1	1	1	1	1	2
*BB28b*	1	1	5	1	2	6	2	3	9
Mean	1.5	2.125	3	1.375	2	2.125	1.375	2.375	5.5

The total number of alleles is given for all samples combined and for the samples from the apartment complex separately.

**Fig 4:**
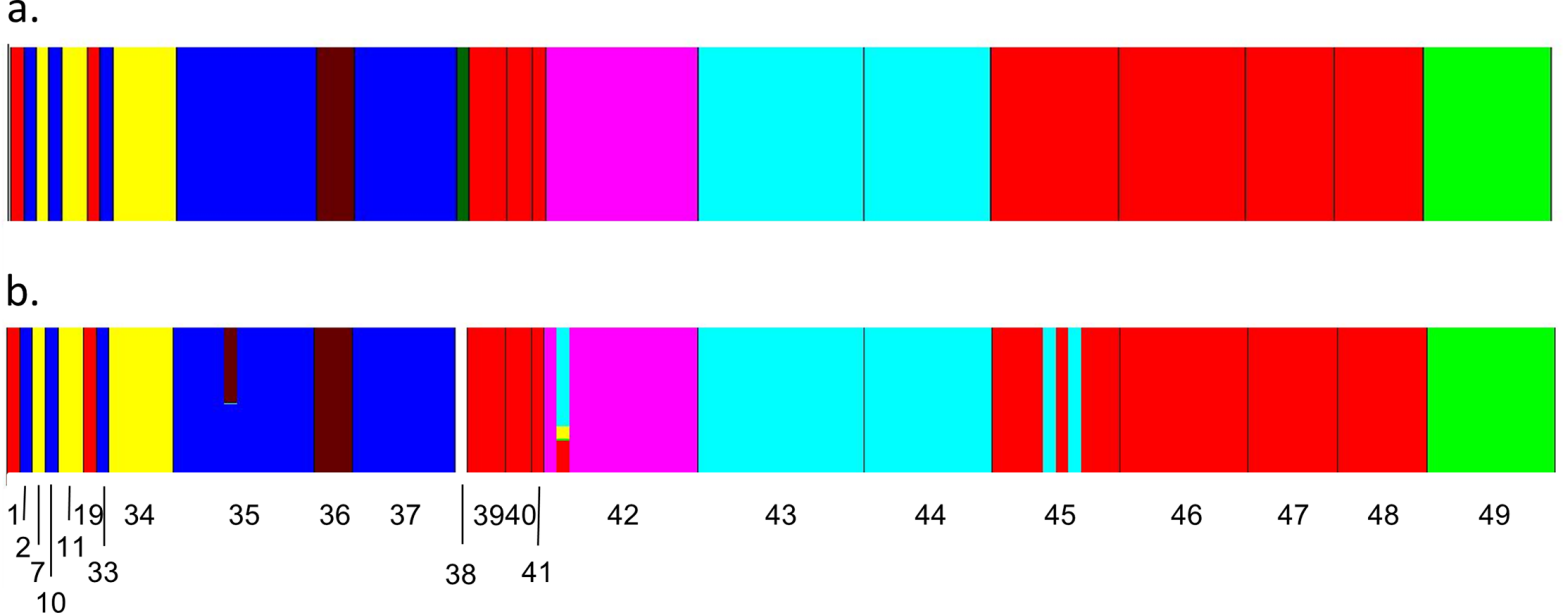
Genetic clusters and degree of admixture identified by the Bayesian clustering method implemented in the program BAPS using only samples collected from the apartment building. (A) The 8 clusters identified in the apartment samples; each individual is shown by a single narrow bar. (B) Admixture of individuals within each cluster with the height of each color proportional to the degree of admixture. Sample 38 was not included in the admixture analysis because it was placed in its own cluster and the minimum population size used in the admixture analysis was 3 individuals. Cluster colors and numbers correspond to those in Fig 5.

**Fig 5:**
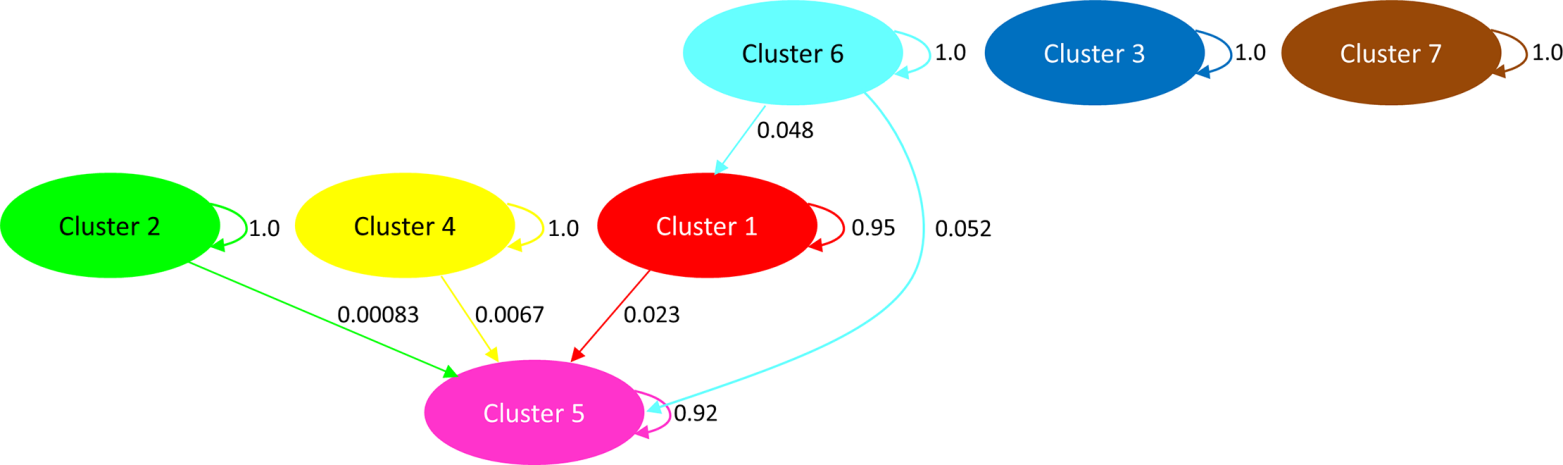
Gene flow diagram among clusters showing the relative amount of ancestry among clusters. The colors for each cluster correspond to the colors shown in Fig 4B. Only samples represented by at least three individuals were included in the admixture analysis.

To obtain a better understanding of substructure within the apartment building, a separate cluster analysis was run using only samples collected from apartments (i.e., excluding the hotel and homeless shelter samples). This analysis also yielded eight clusters, with sample 38, consisting of a single individual, placed in its own cluster (Fig 4A). The admixture analysis showed surprisingly little admixture among clusters (Fig 4B), suggesting little interbreeding or migration among genetically distinct populations. Sample 45 (from apartment 427) had the greatest proportion of individuals showing affiliations with other clusters (samples 43 & 44 from nearby apartment 425), and Cluster 5 (sample 42 from apartment 415) showing one individual with high levels of admixture with both nearby samples 43 & 44 (apartment 425) and samples on other floors. Fig 5 illustrates the presumed level of gene flow among clusters. Clusters 3 (composed of samples 2, 10, 33, 35 and 37) and 7 (sample 36) were the most isolated with no apparent gene flow between them and any other cluster. When analyzed by cluster, populations showed a high degree of genetic differentiation among clusters (*F*_ST_ = 0.431), and were considerably inbred (*F*_IS_ = 0.165) and highly related (*r* = 0.565) within clusters.

Samples 1, 19, 39–41, and 45–48 were genetically similar to one another and illustrate how bed bugs can move between and among apartments (red bars, Fig 4A; red cluster 1, Fig 5). A single nymph (sample 1) and female (sample 19) were collected in June 2012, in units 004 and 327, respectively. Both units were heat-treated about a week later. In December 2012, many nymphs (samples 39 and 40) were collected in unit 325, across from unit 327. Separately, exuviae and eggs (sample 41) were collected in unit 411. Units 323 and 325 were heat-treated, but unit 411 was not, because no live bed bugs were detected. This “red” matriline (cluster 1) was detected again in February 2013 in an infestation of thousands of bed bugs from unit 427 (sample 45); in April 2013, in a heavy infestation of all life stages from unit 005 (sample 46), next to unit 004; one nymph and one adult (sample 47) from unit 325 again; and dozens of blood-fed adults (sample 48) from unit 327 again. We conclude that bed bugs from the same population circulated among units 323, 325, 327, and 427 and also between 004 and 005. These alleles were introduced into other units, such as sample 42 from unit 415, which is adjacent to the laundry facility (Figs 1 and 5). In fact, sample 42 (pink cluster 5 in Fig 5) represents an admixture of itself with clusters 1, 2, 4, and 6 (Figs 4 and 5).

Cluster 6 (aqua, Figs 4A and 5) contributed 4.8% of the alleles in cluster 1 (red) and 5.2% in cluster 5 (pink). Cluster 6 consisted primarily of first and second instar nymphs and two adults (samples 43 and 44), collected in unit 425 in January 2013. Alleles from this population appear in sample 42 from unit 415 and among the thousands of bed bugs from unit 427 (sample 45) in February 2013. Unit 427 is directly above unit 327, which would explain why sample 45 was genetically similar to cluster 1 (units 323, 325, and 327) and across the hall from unit 425, which would explain the contribution of cluster 6 alleles.

On the other hand, clusters 3 and 7 (blue and brown, respectively, Fig 5) appeared as independent introductions with no relation to others. Cluster 3 contained samples 2, 10, 33, 35, and 37, which ranged from the ground floor to the fourth floor. We collected samples 2, 35, and 37 from unit 005 in June, November, and December 2012, respectively. Sample 2 was a single male bed bug under the tenant’s bed. The unit was heat-treated two weeks later. Sample 35 represented a heavy infestation of the blankets and mattress in November. Unit 005 was heat-treated that same day. One month later, the blankets and mattress were again covered with bed bugs genetically similar to the November sample (sample 37, Table 3), indicating the November heat-treatment was ineffective in eliminating the infestation. Unit 005 was heat-treated again one week later. The next sample collection in unit 005 was in April 2013 (sample 46), and it represented a separate matriline, genetically similar to samples 1, 39, 40, and 45–48 from nearby apartment 004 or from units on the third and fourth floors. It thus appears that the December heat-treatment of unit 005 successfully eliminated bed bugs from cluster 3, but bed bugs from an adjacent unit or from the upper floors colonized this ground floor unit between December and April. In contrast, sample 10 was collected in unit 307 in June, and sample 33 in unit 409 in October, reflecting the internal spread of this existing genotype.

Units 306 and 323 were infested in June 2012 with unknown genotypes and heat-treated. Although eggs were collected from unit 323 in September, no live bed bugs were detected until November/December 2012, when both units were infested with new bed bugs representing cluster 7 (brown, unit 306, sample 36) and a unique genotype (dark green, sample 38, Fig 4A) represented by only one individual.

The Bayesian cluster analysis indicated there were 8 distinct genetic groups, suggesting that there were at least 8 separate introductions into the apartment building that successfully established viable populations. Some of these populations spread to other apartment units, whereas others appeared to be confined to a single unit. In the most extreme case, one population appeared to have spread to 6 separate apartments ranging from the ground floor to the fourth floor (Fig 3, pink cluster). The low number of alleles per locus within each cluster—5 of 8 clusters had 4 or fewer alleles at any one locus and the other clusters had at most 5 or alleles at a locus—is consistent with each cluster arising from a single female mated to 1–2 males. The high level of genetic differentiation among clusters (*F*_ST_ = 0.43) and the high relatedness among individuals within clusters (*r* = 0.56) are both consistent with each cluster being a genetically distinct population. Based on these results, we conclude that 16 of 21 infestations for which we had microsatellite data were the result of 3 separate introductions that persisted after heat-treatments and/or spread to additional units, whereas 5 of the infestations arose through separate individual introductions.

Genotypic variation in highly selected traits may not be represented in broad-based genotyping. We found heterogeneity among *kdr* genotypes even between genetically similar populations. For example, samples 35 and 37 were from the same unit and were similar based on microsatellite analysis. We detected five *kdr* genotypes in sample 35, including 4/13 double homozygous resistant mutants, and we found two genotypes in sample 37, including 6/8 double homozygous resistant mutants. Similar heterogeneity in *kdr* genotype frequency was detected between samples 43–44 and 47–48. These values could be the result of re-infestation after heat treatment or migration from other apartments. Because pyrethroid resistance and panmixia are pervasive in this species, insecticides with a novel mode of action are desperately needed. For example, imidacloprid, a neonicotinoid insecticide, irreversibly binds to nicotinic acetylcholine receptors, and studies indicate its potential as part of a bed bug control strategy [32, 33].

We expect our observations regarding infestation/reinfestation patterns and *kdr* genotypes representing pyrethroid resistance will be applicable to bed bug infestations elsewhere. Once a mated female bed bug is introduced into a multi-story building containing hollow, contiguous, vertical chases of concrete, both horizontal and vertical dispersal is facilitated, control is challenging, and elimination requires much more intensive interventions than those conducted here. Heat treatment appears to be effective when conducted concurrently on whole floors or whole buildings and when heat is generated rapidly and given time to penetrate crevices. But a heat intervention might actually encourage dispersal of bed bugs if rooms are not prepared properly, heat is generated slowly, and only individual apartments are treated on as “as needed” basis. Bed bugs find harborage in hollow spaces between units, especially in concrete-lined spaces near the floor that heat up more slowly, and disperse into adjacent units, or they are dispersed by human activity. Moreover, extensive pyrethroid resistance compromises the efficacy of most of the existing pesticides for bed bug control. We conclude that early detection and preventing new introductions is the most cost-effective strategy to thwart bed bug infestations in multi-story apartment buildings. It may be worth housing authorities to consider proactive heat-treatment of furniture in stand-alone heat chambers before tenants move in. This approach circumvents the difficulty of treating concrete structures. Unfortunately, once bed bugs are introduced, they tend to spread and persist in multi-unit housing.
